# Leg reperfusion technique optimization and ischemia diagnosis using ultrasound in patients treated with extracorporeal life support for refractory cardiac arrest – An observational cohort study

**DOI:** 10.1016/j.resplu.2025.101073

**Published:** 2025-08-20

**Authors:** Sebastian Voicu, Sergey Gurevich, Marinos Kosmopoulos, Rajat Kalra, Alejandra Gutierrez, Deborah Jaeger, Tamas Alexy, Bruno Megarbane, Jason Bartos, Demetris Yannopoulos

**Affiliations:** a Division of Cardiology, Department of Medicine, University of Minnesota School of Medicine, Minneapolis, MN, United States; b Center for Resuscitation Medicine, University of Minnesota School of Medicine, Minneapolis, MN 55401, United States; c Assistance Publique Hôpitaux de Paris, Hôpital Lariboisière, Université Paris Cité, INSERM UMR-S 1144, France; d INSERM U 1116, University of Lorraine, Vandœuvre-lès-Nancy, France

**Keywords:** Cardiac arrest, Limb ischemia, Arterial ultrasound, Distal perfusion, Leg ischemia diagnosis

## Abstract

**Objectives:**

Lower limb ischemia after extracorporeal life support for refractory cardiac arrest may be prevented by effective distal perfusion catheter (DPC) placement. We describe the prevalence of cannulated lower limb ischemia, the DPC technique and its improvements over time, and the blood flow velocities by doppler ultrasound associated with ischemia.

**Methods:**

Retrospective single-centre study including patients ≥18 years-old receiving extracorporeal life support under cardiopulmonary resuscitation. Data is expressed as frequencies (percentages), compared using Fisher’s exact test. Logistic regression evaluated technical improvements associated with absence of ischemia. Blood flow velocity cutoffs associated with ischemia were determined according to receiver operator characteristics curves.

**Results:**

We included 338 patients, 60[50–66] years-old. Survival to discharge was 24%.

DPC was placed under ultrasound guidance using stiff wires: initial technique. This was improved resulting in the optimized technique by distal perfusion catheter heparin perfusion, using >12 cm-long sheaths, and using braided sheaths to avoid kinking. Cannulated lower limb ischemia occurred in 23/338 [6.8 %] in the overall population, in 10/77[13 %] with the initial technique and 13/261[5 %] with the optimized technique, p = 0.01. Braided sheaths were associated with absence of ischemia in multivariable analysis, odds ratio 0.18, 95 % confidence interval [0.045–0.65], p = 0.001. Blood flow velocity associated with ischemia was ≤17 cm/s in the mid-superficial femoral artery, and ≤12 cm/s in the more distal arteries.

**Conclusions:**

Using the optimized distal perfusion catheter technique, ischemia prevalence was 5 %. Braided catheters were associated with absence of lower limb ischemia. Blood flow velocity thresholds associated with cannulated lower limb ischemia were ≤17 cm/s.

## Highlights

• Optimized distal perfusion technique decreases leg ischemia in extracorporeal life support.
• Using the optimized distal perfusion technique, ischemia prevalence was as low as 5%
• Use of braided catheters was significantly associated with absence of leg ischemia.
• Blood flow velocities cutoffs associated with cannulated leg ischemia were ≤17 cm/s.

## Graphical abstract

**Figure:**
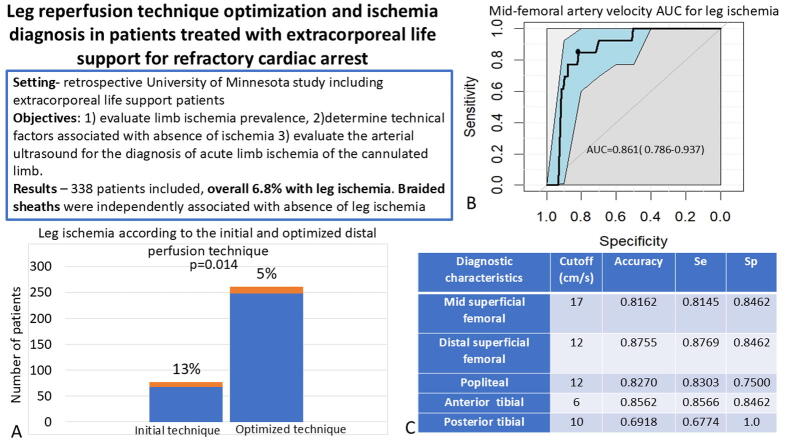

## Introduction

Extracorporeal life support (ECLS) improves survival in refractory cardiac arrest,^1^, ^2^ but requires peripheral cannulation of the femoral artery and vein with large bore cannulas. Both cannulas are problematic as the venous cannula can reduce venous blood return creating venous congestion while the arterial cannula reduces and sometimes completely obstructs antegrade arterial flow into the cannulated limb.^3^ Antegrade distal perfusion of the cannulated limb using a distal perfusion catheter (DPC) can attenuate limb ischemia. However, placement and design of the reperfusion catheter play a role in ischemia which can occur in variable proportions 8–31 %,^4^, ^5^, ^6^, ^7^, ^8^, ^9^, ^10^, ^11^ according to the individual characteristics and management of each cohort. In these reports, the technique of the reperfusion catheter placement was insufficiently described, including technical issues and the material used to avoid specific complications.

Although arterial ultrasound plays an important role in diagnosing leg ischemia in patients not receiving ECLS treatment,^12^, ^13^ the contribution of the ultrasound in the diagnosis of the acute ischemia of the cannulated leg in patients treated with ECLS are insufficiently explored. Furthermore, in the absence of specific diagnostic criteria for ischemia, ultrasound may be underused. In some patients, adequate perfusion of the cannulated leg may depend exclusively on the DPC, and ischemia may rapidly occur if DPC flow is compromised. In these patients, arterial ultrasound may play a role in the early detection of ischemia.

In this study, we evaluated patients receiving ECLS for refractory cardiac arrest from the Minnesota Mobile Resuscitation Consortium (MMRC).^14^ The key objectives of the study were to: 1) evaluate limb ischemia prevalence, 2) describe the evolution of the DPC technique to attenuate limb ischemia, and determine technical improvements independently associated with absence of ischemia 3) evaluate the utility of arterial ultrasound in the diagnosis of acute limb ischemia of the cannulated limb during ECLS.

## Methods

This was a retrospective single-center study including patients receiving ECLS under cardiopulmonary resuscitation (CPR) for refractory cardiac arrest. The study was performed according to the World Health Organization declaration of Helsinki, 2013 version, and received approval from the University of Minnesota Institutional Review Board (IRB approval: 1703M11301). The study used the STROBE cohort reporting guidelines.^15^ Data collection was performed according to the Utstein template.^16^

### Patients

We included patients ≥18 years old receiving ECLS under CPR between December 8th, 2015 and October 1st, 2023, by the MMRC team.^14^ To allow adequate time for manifestation of limb ischemia, we included ECLS patients that survived for at least 6 h after cannulation. We excluded patients who refused participation in retrospective studies.

### Cannulation for extracorporeal life support and management of the patients

The management of the patients with refractory cardiac arrest included ECLS and specialized post-resuscitation care as previously described.^1^, ^14^ After 3 defibrillation attempts without return of spontaneous circulation and in the absence of severe comorbidity, the patient was considered an ECLS candidate.^17^ Cannulation of the vein and artery was performed by the MMRC team using heparin-coated cannulas ranging 21–29 F for veins and 15–19 F for arteries. Ultrasound guidance was used for direct visualization of the needle entering the vessel.^3^ Wire position was confirmed under fluoroscopy. Cannulas were connected to a CardioHelp circuit with a centrifugal pump (Maquet Rotaflow, Maquet Cardiovascular, LLC, Wayne, New Jersey). In most cases an intra-aortic balloon pump was placed in the contralateral artery for ventricular unloading in the absence of pulsatility >10 mmHg.^17^ After cannulation, a bolus of 100 IU/kg of unfractionated heparin was administered followed by continuous infusion for anti Xa target of 0.3–0.5 units/mL unless active bleeding or severe thrombopenia were present. After ECLS initiation, patients were transferred to the cardiac intensive care unit at the University of Minnesota Medical Center for further management.^18^, ^19^

### Distal perfusion catheter placement and definition of the initial and optimized techniques

The reperfusion catheter insertion was adapted over time to address the encountered difficulties and minimize complications. Although the refinements in our technique were sequential over time, we divided the study period into two periods to allow comparisons: 1) the initial technique and 2) the optimized technique. The optimized technique is defined by the last improvement in the process.

**The initial technique** period started with the first included patient in December 2015. A DPC was placed as soon as possible after cannulation, if clinically indicated, by the performing operator. A DPC placement was not considered mandatory and was not performed if the operator considered it was not necessary due to relatively large diameter femoral arteries and good flow of the ECLS. The DPC was placed by superficial femoral artery puncture under vascular ultrasound using a 21 G needle, followed by insertion of a 4 Fr micropuncture sheath over a wire by the modified Seldinger technique. An 0.035 in. Amplatz Extra Stiff^TM^ (Boston Scientific, Boston, MA) wire was advanced into the artery. Over the Amplatz wire, a 9F Arrow two-lumen catheter 10 cm long (Teleflex Medical, Morrisville NC, USA) was inserted as DPC into the superficial femoral artery and connected to the ECLS circuit for leg perfusion. The position of the DPC was confirmed by oxygenated blood return, ultrasound and/or contrast injection under fluoroscopy.

**The optimized technique** consists in placing a DPC in all patients if considered technically feasible. The steps until the insertion of the Amplatz Extra Stiff^TM^ are identical to the initial technique. However, a Super Arrow Flex® braided 7F or 8F sheath (Teleflex Medical, Morrisville NC, USA) 12 or 24 cm long according to the subcutaneous tissue thickness and the BMI of the patient, was used as DPC. The puncture for the DPC placement was performed at a median of 8(6–10) cm distal to the insertion point of the arterial cannula.

### Leg ischemia and vascular complications definitions

Presence of pallor, cyanosis, and distal arterial Doppler using a point-of care device and near-infrared reflectance spectroscopy, were all monitored to determine limb ischemia at 30-minute intervals. Oxygen saturation and creatine kinase levels were used to confirm ischemia or if other signs were inconclusive. Late signs such as mottling, rigidity, and skin lesions were used, but not required for diagnosis due to the late presentation. Bleeding at the cannulation and DPC sites was defined according to the GUSTO classification as severe or life threatening if it caused hemodynamic compromise and required intervention, moderate if it required blood transfusion but did not result in hemodynamic compromise, and mild if it did not require transfusion and was not associated with hemodynamic compromise.^20^

### Use of ultrasound to evaluate flow and diagnose ischemia in the cannulated leg

A baseline assessment of flow of the lower limbs using arterial ultrasound was routinely performed after ICU admission by certified ultrasound technicians and interpreted by accredited radiologists. Measurements were performed on exposed areas of the lower extremities and consisted of maximum flow velocity. In case of suspected ischemia, an ultrasound was repeated in the ischemic limb unless considered unnecessary.

### Statistical analysis

A sample size calculation was not performed in this exploratory study. Continuous variables were expressed as mean or median (Interquartile range 25–75 IQR) and categorical variables as frequencies (percentages). Continuous variables were compared between groups using student *t*-test for normally distributed variables or with Mann-Whitney test if distribution was not normal. Categorical variables were compared using chi-square test of Fischer exact test as appropriate.

Prevalence of leg ischemia and other complications were compared between periods. The sequential improvements in the DPC technique, i.e. heparin infusion in the DPC, length of the DPC and the use of a braided sheath were introduced in univariable logistic regression. Since all improvements were considered clinically relevant to the DPC technique, they were introduced in a multivariable logistic regression model to determine which remained independently associated with limb ischemia.

Diagnostic characteristics of the ultrasound velocities for the diagnosis of acute ischemia in the superficial femoral, popliteal, posterior tibial and anterior tibial arteries, were determined by constructing the receiver operator characteristics (ROC) curves and determining sensitivity, specificity, and the point of maximum accuracy corresponding to the velocity threshold associated with ischemia. No imputations were performed for missing values. Statistical analysis was performed using R statistical software, R version 4.2.2, R Core Team (2022). R: A language and environment for statistical computing. R Foundation for Statistical Computing, Vienna, Austria. URL https://www.R-project.org/.

## Results

**Fig. 1:**
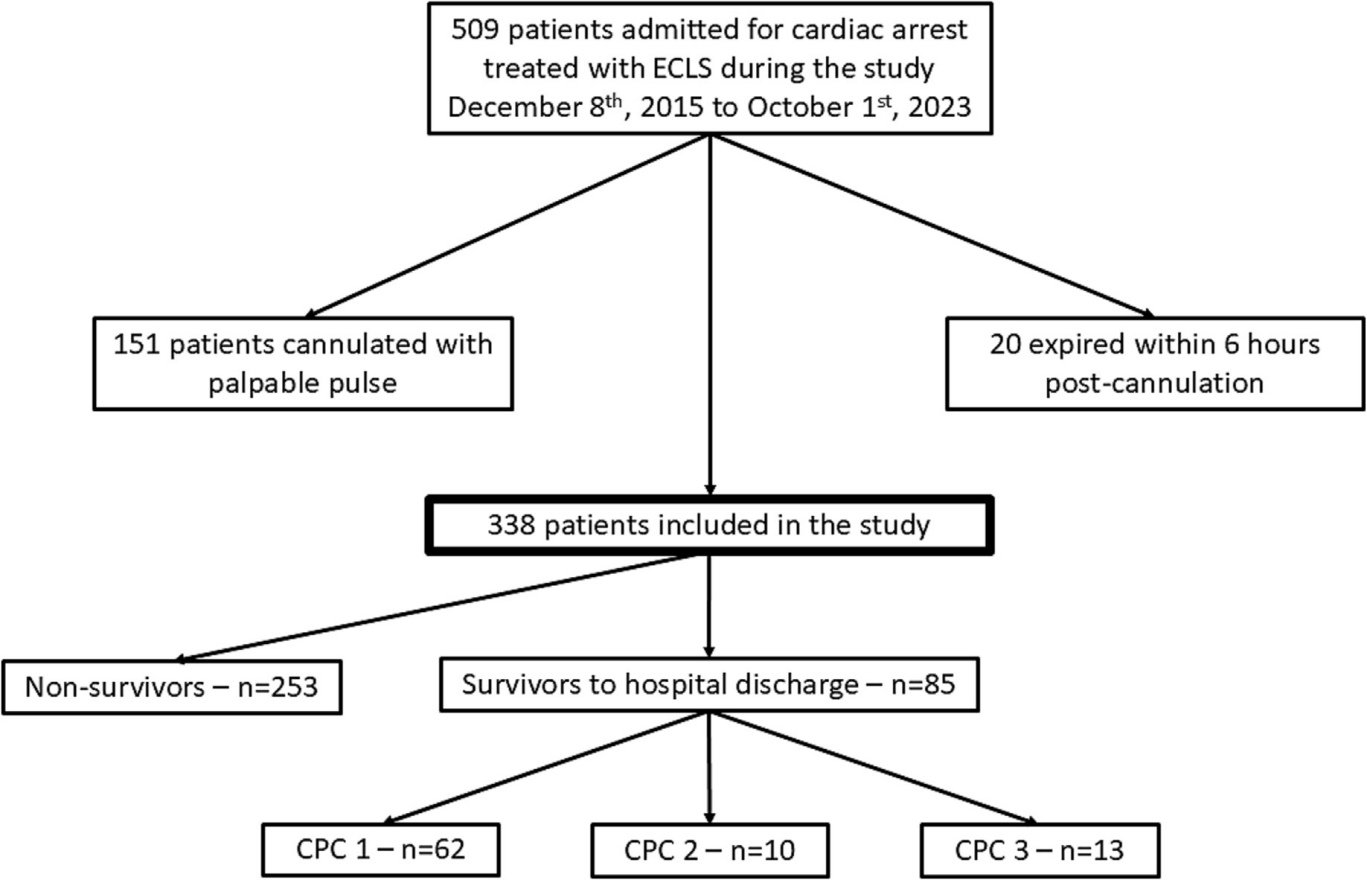
Flow chart of the study. CPC – cerebral performance category.

Three hundred and thirty eight patients (Fig. 1) were included, age 60 (50–66) years old, 96 % had a shockable initial rhythm and 91 % had an out-of-hospital cardiac arrest (Table 1). The time interval between the beginning of CPR and ECLS was 60 (50–72) min. Survival to hospital discharge with Cerebral Performance Category^21^ (CPC) 1 or 2 or 3 was 24 %.

**Table 1:** Characteristics of the patients, management and outcome.

**Characteristic**	**Overall, N** = **338**^a^	**Initial reperfusion technique, N** = **77**^a^	**Optimized reperfusion technique, N** = **261**^a^	**p-value**^b^
Age (years)	60 (50–66)	58 (50–65)	60 (50–66)	0.65
Male gender	270 (80 %)	60 (78 %)	210 (80 %)	0.62
Body mass index(kg/m^2^) (n = 335)	30 (26–34)	30 (26–34)	31 (27–35)	0.29
History of coronary artery disease (n = 337)	77 (23 %)	20 (26 %)	57 (22 %)	0.54
Diabetes mellitus (n = 331)	84 (25 %)	20 (26 %)	64 (25 %)	0.93
Hypertension (n = 330)	142 (43 %)	34 (44 %)	108 (43 %)	0.81
Hypercholesterolemia (n = 328)	111 (34 %)	27 (35 %)	84 (33 %)	0.81
Tobacco smoking (n = 330)	101 (31 %)	16 (21 %)	85 (34 %)	0.03
Delay arrest to EMS arrival (n = 352)	5 (4–10)	6 (5–10)	5 (3–10)	0.02
Delay EMS on site to hospital/cathlab (min) (n = 268)	31 (21–40)	36 (23–42)	30 (20–40)	0.08
Arrest location (n = 332)				0.70
Home	203 (61 %)	44 (58 %)	159 (62 %)	
Public place	100 (30 %)	26 (34 %)	74 (29 %)	
Hospital	29 (9 %)	6 (8 %)	23 (9 %)	
Initial rhythm (n = 337)				0.70
Shockable	324 (96 %)	75 (97 %)	249 (96 %)	
Non shockable	13 (3.9 %)	2 (2.6 %)	11 (4.2 %)	
Witnessed	270 (80 %)	55 (71 %)	215 (82 %)	0.35
Bystander CPR (n = 335)	240 (72 %)	51 (66 %)	189 (73 %)	0.20
Intubation by EMS (n = 332)	207 (62 %)	56 (73 %)	152 (60 %)	0.07
Time interval from CPR to ECLS (min) (n = 293)	60 (50–72)	65 (57–75)	58 (50–70)	0.006
Plasma lactate on arrival (mmol/L) (n = 318)	12.3 (9.8–15.0)	12.5 (10.6–15.0)	12.0 (9.8–14.9)	0.40
Arterial pH (n = 314)	7.00 (6.89–7.11)	7.02 (6.92–7.15)	7.00 (6.88–7.10)	0.20
Arterial P_CO2_ on arrival (n = 304) (mmHg)	50 (36–67)	45 (37–62)	51 (35–69)	0.30
Arterial P_O2_ on arrival (n = 308) (mmHg)	113 (61–138)	104 (60–131)	116 (63–141)	0.20
Plasma bicarbonate on arrival mmol/L (n = 383)	16.0 (13.0–19.0)	16.0 (13.0–19.0)	16.0 (13.0–19.0)	0.95
Insertion of IABP(n = 335)	224 (67 %)	47 (61 %)	177 (69 %)	0.20
Impella® insertion (n = 333)	4 (1.2 %)	3 (3.9 %)	1 (0.4 %)	0.04
Survived decannulation (n = 336)	124 (37 %)	34 (44 %)	90 (35 %)	0.13
Duration of ECLS treatment (days)	3 (2–5)	3 (2–5)	3 (2–5)	0.95
Cause of arrest cardiac ischemia (n = 336)	229 (68 %)	52 (68 %)	177 (68 %)	0.90
Coronary revascularization	188 (56 %)	47 (61 %)	141 (54 %)	0.30
Survived to discharge	92 (27 %)	26 (34 %)	66 (25 %)	0.14
CPC at discharge				0.06
1	62 (18 %)	17 (22 %)	45 (17 %)	
2	10 (3 %)	1 (1 %)	9 (4 %)	
3	13 (4 %)	5 (7 %)	8 (3 %)	
4	7 (2 %)	4 (5 %)	3 (1 %)	
5	246 (73 %)	50 (65 %)	196 (75 %)	

a n (%); Median (IQR).

b Fisher's exact test; Pearson's Chi-squared test; Wilcoxon rank sum test; EMS-emergency medical service; CPR-cardiopulmonary resuscitation; ECLS-extracorporeal life support; IABP – intraaortic balloon pump; CPC-cerebral performance category; n in parentheses in the “Characteristics” column represents the number of patients with available data. For parameters where no n = is specified, data was available in all patients.

### Technical improvements in reperfusion catheter placement and the two periods of the study

In November 2016 we decided to routinely place a DPC in all patients after a high rate of limb ischemia (4 out of 8) was noted in patients treated without a DPC. Following a case of distal perfusion catheter thrombosis, on January 2018, we decided to perfuse additional heparin into the DPC at 2 ml/hour (10 IU/ml) using a three-way stopcock. Initially, a 9F Arrow two-lumen catheter 10 cm long (Teleflex Medical, Morrisville NC, USA) was used as DPC but since July 2018, it was decided to place long sheaths (24 cm) after the DPC was inadvertently removed in 2 patients with BMI > 30. The decision to implement braided sheaths in all patients was made on November 2018, after a reperfusion catheter was found kinked and obstructed. This it is the last improvement of the DPC technique and separates the initial technique period from the optimized technique period of the study.

### Leg ischemia prevalence and technical factors associated with absence of leg ischemia

**Fig. 2:**
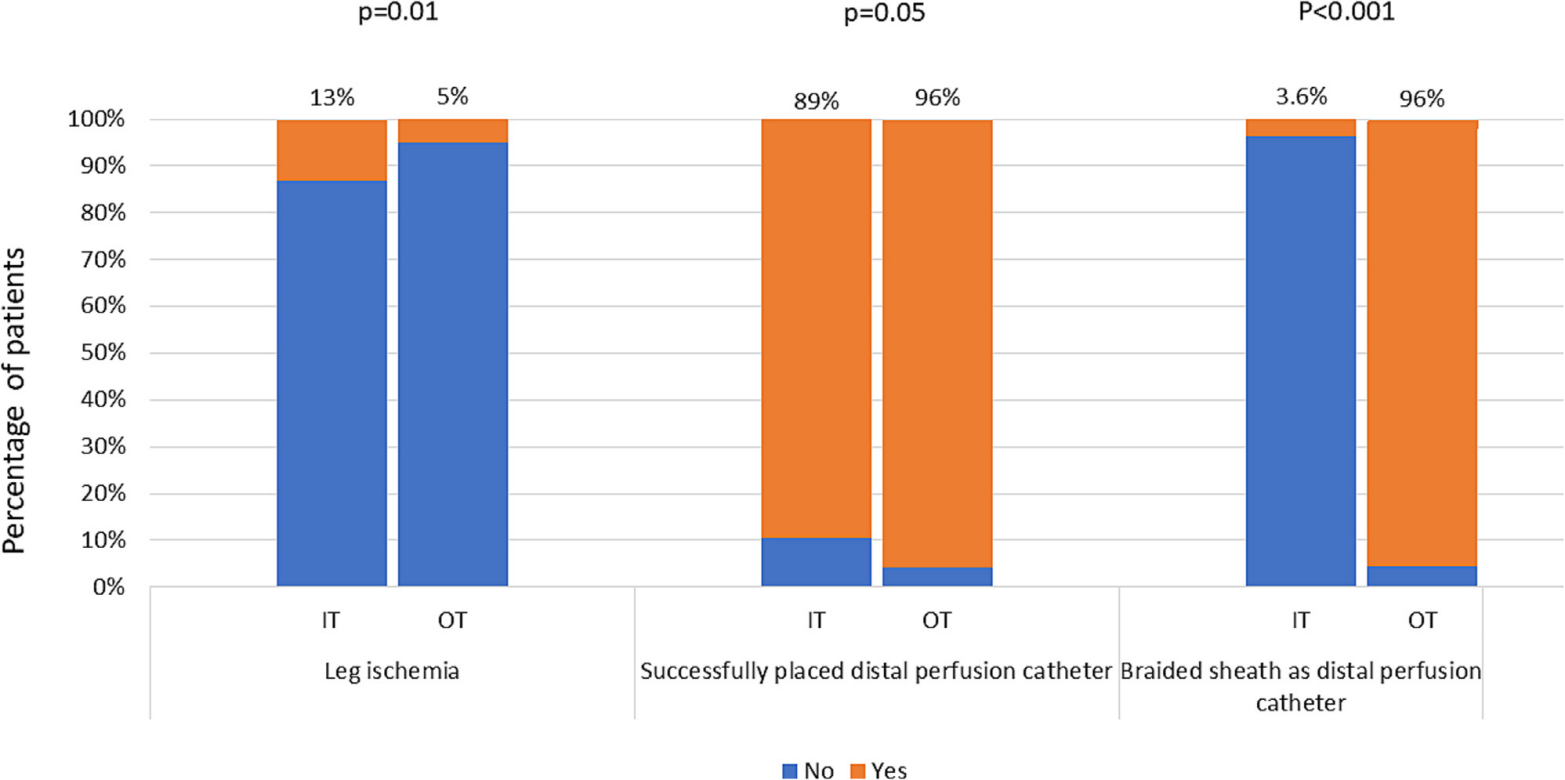
Leg ischemia, placement of a distal perfusion catheter, and use of braided sheath according to the distal perfusion technique period*.* IT- initial technique period, OT – optimized technique period.

Leg ischemia occurred overall in 23 (6.8 %) patients, 10(13 %) in the initial technique and 13 (5 %) in the optimized technique period, p = 0.014, Table 2, Fig. 2. Time to leg ischemia observed by ultrasound was 15 (11–22) hours post cannulation. In the initial period, placing a DPC was not attempted in 8 patients because it was not considered necessary according to the operators in charge and 4 (50 %) developed ischemia. In the rest of the study, it was not placed in 11 additional patients, deemed not feasible or too prone to complications due to severe peripheral artery disease, and 5 among them (45 %) developed ischemia. With the optimized technique, out of the 250 patients in whom a DPC was feasible and was successfully placed, only 8 (3.2 %) developed cannulated leg ischemia.

**Table 2:** Characteristics of the reperfusion technique and complications.

**Characteristic**	**Overall**, N = 338^a^	**Initial reperfusion technique**, N = 77^a^	**Optimized reperfusion technique**, N = 261^a^	**p-value**^b^
Arterial cannula size (French, n = 337)				0.007
15	72 (21.4 %)	24 (31 %)	48 (18 %)	
17	257 (76.3 %)	49 (64 %)	208 (80 %)	
19	8 (2.3 %)	4 (5 %)	4 (2 %)	
Venous cannula size (French, n = 337)				0.80
21	1 (0.3 %)	0 (0 %)	1 (0.4 %)	
23	4 (1.2 %)	0 (0 %)	4 (1.5 %)	
24	2 (0.6 %)	0 (0 %)	2 (0.8 %)	
25	324 (96 %)	75 (97 %)	249 (96 %)	
27	3 (0.9 %)	1 (1.3 %)	2 (0.8 %)	
29	3 (0.9 %)	1 (1.3 %)	2 (0.8 %)	
Successfully placed reperfusion catheter (n = 337)	318 (94 %)	68 (89 %)	250 (96 %)	0.05
Reperfusion catheter not attempted (n = 335)	19 (5.7 %)	9 (12 %)	10 (3.8 %)	0.02
Caliber of the reperfusion catheter (n = 311)				<0.001
6	3 (1 %)	0 (0 %)	3 (1 %)	
7	26 (8 %)	0 (0 %)	26 (11 %)	
8	205 (66 %)	1 (2 %)	204 (83 %)	
9	77 (25 %)	65 (98 %)	12 (5 %)	
Long (>12 cm) reperfusion catheter (n = 252)	69 (27 %)	0 (0 %)	119 (61 %)	<0.001
Braided sheath for reperfusion (n = 278)	219 (77 %)	2 (3.6 %)	217 (96 %)	<0.001
Failure to insert reperfusion catheter (n = 317)	7 (2.2 %)	3 (4.3 %)	4 (1.6 %)	0.20
Systemic heparin (n = 336)	315 (94 %)	77 (100 %)	238 (92 %)	0.006
Heparin in reperfusion catheter (n = 325)	258 (79 %)	22 (29 %)	236 (94 %)	<0.001
Cannulas related bleeding (n = 91)^c^				0.70
Severe	12 (13 %)	1 (5.6 %)	11 (15 %)	
Moderate	33 (36 %)	7 (39 %)	26 (36 %)	
Mild	46 (51 %)	10 (56 %)	36 (49 %)	
Reperfusion catheter-related bleeding^c^ (n = 15)				0.50
Severe	2 (13 %)	2 (29 %)	0 (0 %)	
Moderate	5 (33 %)	2 (29 %)	3 (38 %)	
Mild	8 (53 %)	3 (43 %)	5 (63 %)	
Hematoma at the cannulation site	43 (13 %)	10 (13 %)	33 (13 %)	0.95
Hematoma at the distal perfusion catheter	6 (1.8 %)	3 (3.9 %)	3 (1.1 %)	0.13
Number of red blood cell packs transfused (n = 250)	9 (3–11)	9 (1–11)	9 (5–11)	0.40
Ischemia of the cannulated leg	23 (6.8 %)	10 (13 %)	13 (5.0 %)	0.01
Ischemia of the contralateral (IABP) leg	13 (3.8 %)	1 (1.3 %)	12 (4.6 %)	0.30
Arterial dissection (n = 334)	3 (0.9 %)	0 (0 %)	3 (1.2 %)	0.95
Fasciotomy	4 (1.2 %)	2 (2.6 %)	2 (0.8 %)	0.20
Vascular surgery	12 (3.6 %)	4 (5.2 %)	8 (3.1 %)	0.50
Vascular interventional procedure (n = 336)	2 (0.6 %)	0 (0 %)	2 (0.8 %)	0.95
Creatine phosphokinase peak (Units/L)	2,492 (909–5,475)	3,633 (2,008–6,641)	2,208 (832–5,136)	0.12

a n (%); Median (IQR).

b Fisher's exact test; Pearson's Chi-squared test; Wilcoxon rank sum test.

c According to the GUSTO classification; IABP – intraaortic balloon pump; n in parentheses in the “Characteristics” column represents the number of patients with available data. For parameters where no n = is specified, data was available in all patients.

Improvements to the technique assessed in univariable analysis were: heparin infusion in the DPC, placement of a long (24 cm) versus short (≤12 cm) DPC, and use of a braided sheath as DPC (Table 2). Given the use of 21 G needles, ultrasound guidance for access and Amplatz stiff wires in all patients, these factors could not be assessed in univariable analysis. Heparin infusion in the DPC and length of the catheter were not associated with absence of ischemia, OR = 0.508 (0.159–1.928), p = 0.27, and OR = 1.358 (0.379–5.425), p = 0.64 respectively. The use of a braided sheath was significantly associated with absence of ischemia, OR = 0.18 (0.045–0.65), p = 0.001.

In multivariable analysis, only the use of a braided sheath was significantly associated with absence of ischemia, OR = 0.054 (0.002–0.533), p = 0.03 (more detailed results are provided in the Supplemental data). Ischemia in the 13 non-cannulated legs was due to IABP in 11 patients and to an arterial line in 2 patients (Table 3).

**Table 3:** Ultrasound data in the cannulated and non-cannulated legs, at baseline and during the ischemia episode.

**Cannulated leg data**	**Overall N = 338**	**Baseline ultrasound patients without cannulated leg ischemia**^a^**N = 315**	**Ultrasound during the leg ischemia, patients with cannulated leg ischemia**^a^**N = 23**	
Mid-SFA systolic velocities (cm/s) (n = 283)	39 (19–65)	43 (20–69)	0 (0–16)	<0.001
Distal SFA systolic velocities (cm/s) (n = 271)	28 (15–44)	30 (17–45)	7 (0–11)	<0.001
Retrograde flow in the femoral artery (n = 272)	16 (5.9 %)	16 (6.2 %)	0 (0 %)	0.95
Popliteal systolic velocities (cm/s) (n = 288)	21 (13–35)	22 (14–37)	8 (1–16)	<0.001
Anterior tibial artery at the ankle (cm/s) (n = 291)	12 (7–24)	12 (8–26)	0 (0–5)	<0.001
Posterior tibial at the ankle (cm/s) (n = 292)	15 (7–31)	17 (8–32)	4 (0–8)	<0.001
Time interval from cannulation to ultrasound (hours, n = 279)	11 (6–16)	10 (6–16)	15 (11–22)	0.03
Arterial cannula size (French, n = 337)				0.20
15	72 (21 %)	65 (21 %)	7 (30 %)	
17	257 (76 %)	242 (77 %)	15 (65 %)	
19	8 (2.4 %)	7 (2.2 %)	1 (4.3 %)	

**Non cannulated leg data**	**Overall N = 338**	**Baseline ultrasound, patients without non-cannulated leg ischemia**^a^**N = 325**	**Ultrasound during the leg ischemia, patients with non-cannulated leg ischemia**^a^**N = 13**	
Mid-SFA systolic velocities (cm/s) (n = 284)	54 (37–74)	55 (38–74)	11 (5–29)	0.008
Distal SFA systolic velocities (cm/s) (n = 287)	42 (26–63)	43 (26–63)	14 (4–29)	0.02
Popliteal systolic velocities (cm/s) (n = 287)	35 (21–52)	36 (21–52)	13 (8–47)	0.05
Anterior tibial artery at the ankle (cm/s) (n = 291)	24 (12–41)	24 (14–42)	3 (0–6)	<0.001
Posterior tibial at the ankle (cm/s) (n = 292)	27 (14–44)	28 (15–46)	1 (0–7)	<0.001
Time interval from cannulation to ultrasound (hours, n = 279)	11 (6–16)	11 (6–16)	7 (5–12)	0.06
Intraaortic balloon pump (n = 335)	224 (67 %)	213 (66 %)	11 (85 %)	0.20

a n (%); Median (IQR). ^b^Fisher's exact test; Pearson's Chi-squared test; Wilcoxon rank sum test; SFA-superficial femoral artery; n in parentheses in the “Characteristics” column represents the number of patients with available data. For parameters where no n = is specified, data was available in all patients.

### Other complications

Bleeding at the site of the DPC occurred in 15 (4.4 %) of the patients, and at the cannulation site in 91 (27 %) including mild bleeding/hematoma and bleeding not clinically apparent, diagnosed by post-cannulation CT-scan. Transfusion was necessary in 7 (2 %) patients for DPC bleeding and in 45 (13 %) for cannulation bleeding, during the period from cannulation to 24 h post-decannulation (Table 2). Vascular surgery was required in 3.6 % of the cases, fasciotomy in 1.2 %, and vascular interventional procedures in 0.6 % of the cases (Table 2). There were no amputations.

### Leg ultrasound and blood flow velocities for cannulated leg ischemia diagnosis

**Fig. 3:**
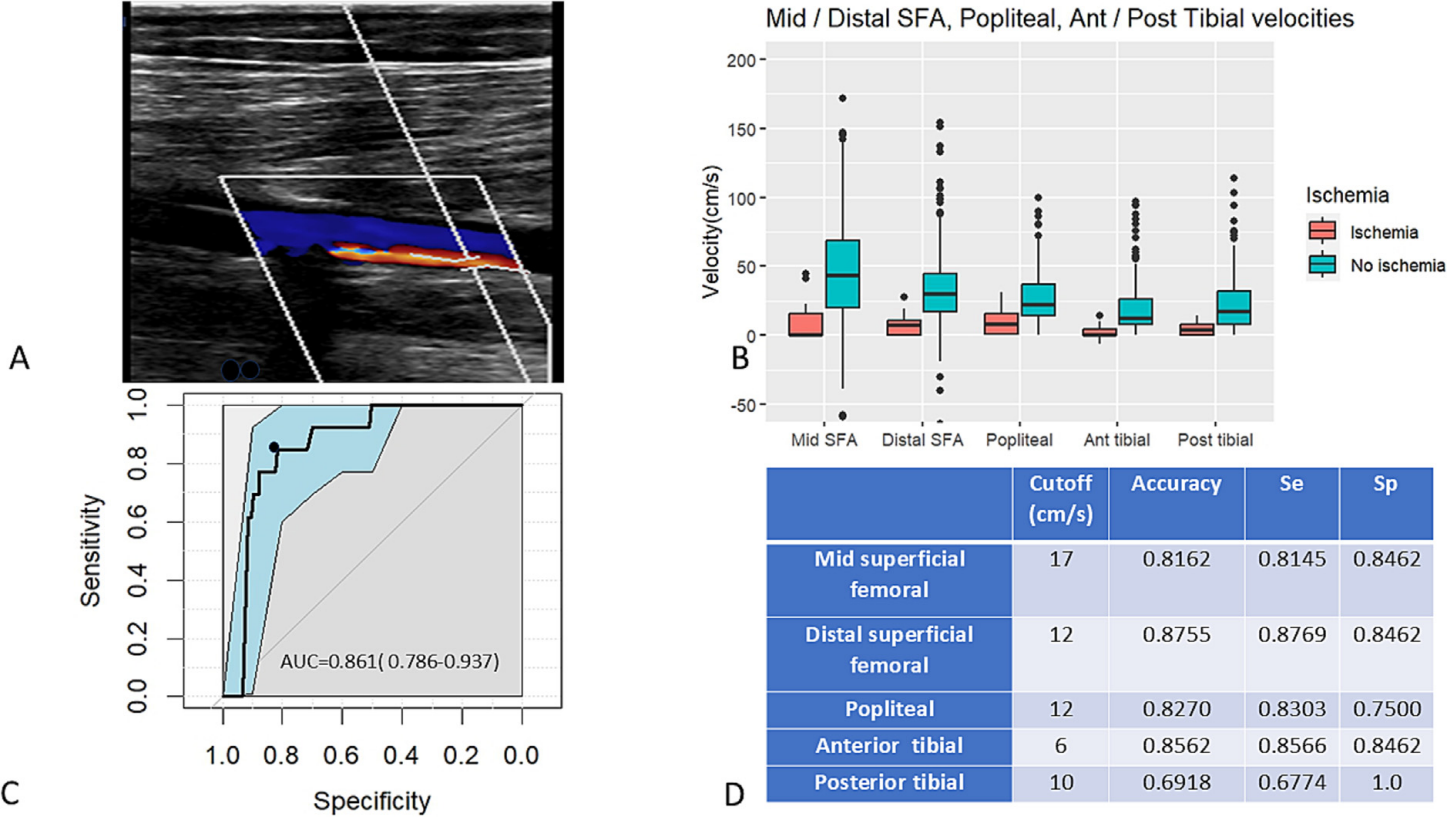
Systolic velocities for the diagnosis of cannulated leg ischemia. A − reversed blood flow in the superficial femoral artery (in blue). The flow inside the DPC (distal perfusion catheter) is antegrade and is figured in red and the reverse flow outside the DPC in blue. B – peak systolic blood flow velocities in the arteries of the lower limbs with and without ischemia, and corresponding diagnostic characteristics. C- Receiver operator characteristics curve for SFA systolic velocities of the cannulated leg. The black dot represents the point of maximum accuracy. D – diagnostic characteristics of cutoffs for leg ischemia diagnosis in the cannulated leg. SFA − superficial femoral artery, Ant − anterior, Post − posterior, Se − sensitivity, Sp – specificity. (For interpretation of the references to colour in this figure legend, the reader is referred to the web version of this article.)

A leg ultrasound examination was performed in 300 (89 %) of the patients. Ultrasound data are expressed in Table 3 showing the comparison between patients who did not develop ischemia *versus* patients who developed ischemia. According to ROC curves analysis, the cut-off for blood flow velocity in the mid superficial femoral artery associated with leg ischemia was ≤ 17 cm/s, and between 6 and 12 cm/s in the rest of the arteries of the cannulated leg (Fig. 3B, C, D).

## Discussion

The incidence of ischemia of the cannulated leg was remarkably low, 5 %, with the optimized technique in this cohort of patients receiving ECLS under CPR for refractory cardiac arrest. In patients without severe peripheral artery disease in whom a DPC was successfully placed, this prevalence was even lower, 3.2 %. This compares favourably to previous studies reporting ischemia rates between 7.7 %^4^ and 31 %^11^ in mixed cohorts of refractory arrest and cardiogenic shock patients. The present study is the first to include a homogenous population of patients cannulated percutaneously under CPR which, together with the absence of pulse, pose specific challenges for the percutaneous vascular access.

The use of a braided sheath as the DPC was associated with reduced ischemia in the cannulated limb. This type of sheath has the advantage of increased radial strength and is less prone to kinking-related occlusion. Heparin infusion in the DPC and the use of long sheaths were not associated with less cannulated leg ischemia, either because they may be ineffective, or due to their implementation from a relatively early stage in the study reducing the odds of finding a significant association with the endpoint. These data indicate that if one measure is implemented it would be reasonable that this measure be the use of braided sheaths as DPC.

For limb ischemia diagnosis, absence of flow is indicative of acute ischemia and can be used to diagnose ischemia in the non-ECLS^12^ and ECLS patients,^22^ while other parameters like dampened distal flow^12^ are used in patients not supported by ECLS. In patients not supported by ECLS, the ratio of the systolic velocities upstream and downstream of the stenosis is used for ischemia diagnosis, with cut-offs >2 or >2.5 or >3.^23^, ^24^, ^25^ In ECLS patients, these criteria cannot be employed due to non-physiologic continuous flow from the ECLS cannula with severely reduced or absent pulsatility and dampened flows downstream of the arterial cannula. We therefore focused on the maximum velocities as diagnostic criteria and determined diagnosis cut-offs which have not been previously investigated. A previous study documented ultrasound velocities in the superficial femoral artery in 19 patients supported by ECLS, but no ultrasound was performed during the ischemic episodes and cutoffs/thresholds for ischemia were not determined.^26^

Interestingly, we observed flow reversal in the parts of the superficial femoral artery proximal to the tip of the DPC in patients without ischemia. Therefore, reversed flow in the superficial femoral artery should not be considered a sign of lower limb ischemia but only a sign that perfusion of the thigh is heavily reliant on the DPC flow due to the arterial cannula obstruction of most or all antegrade flow. The placement site of the reperfusion catheter is variable across studies: the common femoral artery^4^ or the superficial femoral artery.^6^, ^27^ In both techniques, the tip of the DPC lies in the superficial femoral artery distal to the entry point of the arterial cannula and retrograde flow may occur.

Other complications like bleeding were reported in our cohort with a relatively high prevalence, because we also reported minor and non-clinically significant bleeding. Only 45 (13 %) of the patients received transfusion due to bleeding related to the ECLS cannulas and 7 (2 %) due to DPC-related bleeding during their hospitalization. Although bleeding in other cohorts was not reported according to specific classifications as was the case in the present study, bleeding in our cohort compares favourably with previous data reporting up to 9.4 % bleeding in the post-decannulation period alone.^6^

The present study explored the technical aspects of the leg reperfusion procedure and material used but did not evaluate the role of DPC in preventing ischemia because the decision to place a DPC in all patients was made very early during our study due to 50 % incidence of leg ischemia in the 8 patients not receiving a DPC in the initial period, and was supported by *meta*-analyses and reviews of the published data.^28^, ^29^, ^30^ Previous studies focused on this topic showing that routine placement of a DPC was associated with protection against leg ischemia^6^, ^31^ although other authors did not confirm this association.^5^, ^32^

### Limitations

Some of the technical aspects of the leg reperfusion could not be explored in our study due to identical practice in all patients throughout its course, such as the type of needle used for access, the use of ultrasound guidance, and the type of wire used for DPC insertion. It is therefore unclear if these choices are associated with less ischemia or other complication. All patients received intravenous vasopressors which are generally associated with limb ischemia,^33^ but total dose and maximum infusion rate were not available. However, our focus was on the ischemia prevalence, technical factors associated with it and ultrasound diagnostic criteria, therefore analysing vasopressor dose and infusion rate would likely not have influenced our main results. We explored the initial versus optimized technique of DPC placement in our study, but this type of design could not account for the learning curve of the operators. Our investigation was based on the MMRC cohort and some degree of overlap in the included populations may exist with the study by Siems et al, however, the populations differ in the inclusion periods and the patients included: patients with cardiopulmonary failure including those referred from other centers, cannulated by surgical or percutaneous techniques,^5^ *versus* patients with refractory cardiac arrest cannulated percutaneously by the MMRC staff in our cohort. Point-of care and near-infrared reflectance spectroscopy were used for bedside monitoring to detect leg ischemia, however quantitative data for these techniques alerting the clinician to the potential ischemia were not analysed. Data was collected retrospectively leading to missing data for some parameters. Finally, we determined ultrasound velocities in cannulated legs with and without ischemia and suggested velocity thresholds associated with cannulated leg ischemia, but we did not validate these in a different cohort of patients, and this evaluation could be performed in future studies.

## Conclusion

Using the contemporary distal perfusion technique, ischemia was as low as 5 % and the use of braided catheters for distal perfusion was associated with absence of leg ischemia. Blood flow velocity thresholds associated with cannulated leg ischemia were determined by ultrasound examination and could be used for leg ischemia diagnosis in ECLS patients.

## CRediT authorship contribution statement

**Sebastian Voicu:** Writing – review & editing, Visualization, Investigation, Formal analysis, Data curation, Conceptualization. **Sergey Gurevich:** Writing – review & editing, Supervision, Methodology, Investigation, Formal analysis. **Marinos Kosmopoulos:** Writing – review & editing, Validation, Investigation, Formal analysis, Conceptualization. **Rajat Kalra:** Writing – review & editing, Validation, Supervision, Methodology, Investigation, Formal analysis, Conceptualization. **Alejandra Gutierrez:** Writing – review & editing, Validation, Investigation, Formal analysis, Conceptualization. **Deborah Jaeger:** Writing – review & editing, Validation, Investigation, Formal analysis, Data curation, Conceptualization. **Tamas Alexy:** Writing – review & editing, Validation, Methodology, Formal analysis, Conceptualization. **Bruno Megarbane:** Writing – review & editing, Validation, Supervision, Methodology, Formal analysis, Conceptualization. **Jason Bartos:** Writing – review & editing, Validation, Supervision, Resources, Project administration, Methodology, Investigation, Formal analysis, Conceptualization. **Demetris Yannopoulos:** Writing – review & editing, Validation, Supervision, Resources, Project administration, Methodology, Funding acquisition, Formal analysis, Conceptualization.

## Funding

This research received no specific grant from any funding agency in the public, commercial or not-for-profit sectors.

## Declaration of competing interest

The authors declare that they have no known competing financial interests or personal relationships that could have appeared to influence the work reported in this paper.
