# Supplementary material for: Leg reperfusion technique optimization and ischemia diagnosis using ultrasound in patients treated with extracorporeal life support for refractory cardiac arrest – An observational cohort study
Source: Resusc Plus. 2025 Aug 20;26:101073. doi: 10.1016/j.resplu.2025.101073 (PMC12441614; doi:10.1016/j.resplu.2025.101073)
Supplement: Supplementary Data 1 [file mmc1.docx]

Multivariable analysis results for factors associated with cannulated leg ischemia

| Variable | Estimate | Standard error | z-value | p |
| --- | --- | --- | --- | --- |
| Intercept | -0.771 | 1.470 | -0.524 | 0.600 |
| Long sheath | -1.636 | 1.347 | -1.214 | 0.225 |
| Braided sheath | -2.923 | 1.346 | -2.171 | 0.029 |
| Heparin infusion in DPC | 0.256 | 0.821 | 0.312 | 0.755 |

Multivariable analysis odds ratios for ischemia of the cannulated leg

| Variable | Odds ratio | Confidence interval low (2.5) | Confidence interval high (97.5) |
| --- | --- | --- | --- |
| Long sheath | 0.195 | 0.007 | 1.889 |
| Braided sheath | 0.054 | 0.002 | 0.533 |
| Heparin infusion in DPC | 1.292 | 0.263 | 7.219 |
